# Carcass traits and meat quality of lambs fed with cactus *(Opuntia fícus-indica Mill)* silage and subjected to an intermittent water supply

**DOI:** 10.1371/journal.pone.0231191

**Published:** 2020-04-07

**Authors:** Aelson Fernandes do Nascimento Souza, Gherman Garcia Leal de Araújo, Edson Mauro Santos, Paulo Sérgio de Azevedo, Juliana Silva de Oliveira, Alexandre Fernandes Perazzo, Ricardo Martins Araujo Pinho, Anderson de Moura Zanine

**Affiliations:** 1 Department of Animal Science, Universidade Federal da Paraíba, Areia, Brasil; 2 Empresa Brasileira de Pesquisa Agropecuária, Petrolina, Brasil; 3 Department of Animal Science, Universidade Federal do Maranhão, Chapadinha, Brasil; University of Illinois, UNITED STATES

## Abstract

The present study aimed to evaluate the effect of cactus silage and an intermittent water supply for lambs on carcass traits and meat quality. Thirty-six crossbreed lambs with an initial average weight of 19.8 ± 2.1 kg and average age of 6 months were randomly assigned to a 3 × 3 factorial design comprising three addition ratios of cactus silage to the diet (0, 21, and 42% based on dry matter) and three water supply intervals (0, 24, and 48 h) with four replicates. There was no interaction (*P* > 0.05) between the cactus silage ratio and intermittent water supply for any of the evaluated variables, with the exception of the yield of the half carcass commercial cuts. There was no significant effect of intermittent water supply (*P* > 0.05) on the carcass characteristics or meat quality. The addition of forage cactus silage as a substitute for Tifton hay affected the morphometric measurements (*P* < 0.05) and carcass compactness index (*P* < 0.05). The addition of 42% cactus silage to the diet increased (*P* < 0.05) the rib eye area (13.98 cm^2^). The addition of cactus silage as a substitute for Tifton hay positively affected (*P* < 0.05) the carcass weight of commercial cuts of the lambs. To the physical and chemical parameters of the *Longissimus lumborum*, the addition of 42% cactus silage to the diet reduced the color meat parameters (P < 0.05) and pH_0 and 24h_, cooking losses, and shear force were not affected (*P* > 0.05). The addition of forage cactus silage to the lambs’ diet affected (*P* < 0.05) the composition of some saturated fatty acids in the meat. A water supply interval of up to 48 h does not influence carcass characteristics and meat quality. Therefore, the use of cactus silage can be recommended in situations of water scarcity without harming the production or meat quality of crossbreed lambs.

## Introduction

In livestock production, nutrition represents an important factor to improve animal performance and, consequently, improve carcass traits and meat yield to be marketed. However, especially in arid and semi-arid regions, due to irregular rainfall, the availability of animal feed and quality water for animals is the main problem faced by a production system. In these regions, the low precipitation associated the unfavourable geological structures for subsoil water accumulation [1] results in a scarcity of potable water to be supplied to animals, which can compromise feed intake and consequently the animals’ performance.

*Opuntia* is an essential forage for ruminant production in these regions since it is well accepted and consumed by animals, is a feed of excellent palatability, has a high energy value (TDN of 660–740 g/kg), high digestibility (690–780 g/kg), and is rich in water (water content of 926–860 g/kg), therefore contributing to the supply of quality water for the animal [2, 3]. This is important since during the dry season, the lack of water severely limits livestock production, and frequently, herds need to move several kilometres to reach a water source. In such cases, an intermittent water supply can be used as a strategy to mitigate the effects of water scarcity. Several studies [4, 5] have shown that production is not significantly jeopardised by mild or moderate water restriction, constituting an important strategy during the dry season.

Thus, since cactus harvesting and regrowth occur simultaneously, cactus silage is an alternative for the producer in arid and semiarid regions [6]. Although the dry matter (73.8–140 g/kg DM), neutral detergent fibre (210–228 g/kg DM), and crude protein (40–60g/kg DM) levels are lower in cactus silage than in other feed sources commonly used for ruminants, cactus silage represents a good alternative if used together with other, more nutrient-rich roughages, especially when fresh fodder is limited [2, 3], without damaging the performance, carcass traits, and meat quality of the animals.

Studies evaluating the effects of water restriction on carcass characteristics are still incipient and mainly consider the use of wet fodder, such as *Opuntia*, to mitigate the effects of intermittent water supply on carcass traits [3].

Given the above it is hypothesized that the use of cactus silage meets the water demand of animals even when in water intermittent maintaining the characteristics of carcass and meat quality of the animals. The aim of the present study was to evaluate the effect of cactus silage and an intermittent water supply for lambs on carcass traits and meat quality.

## Material and methods

### Ethical approval

The experiment was approved by the Ethics Committee on Animal Use of the Federal University of Bahia (Protocol number 04/2016), in accordance with the guidelines of the National Council for the Control of Animal Experimentation (Brasilia, Brazil; CONCEA).

### Study area

The experiment was conducted in the Animal Nutrition sector of the Embrapa Semiárido (Brazilian agricultural research agency), located in the municipality of Petrolina, PE, Brazil, at 9°23'35” S and 40°30'27" W.

### Animals and feed management

A total of 36 crossbreed lambs, with an initial average weight of 19.8 ± 2.1 kg and an average age of 6 months were used. Prior to the experiment, the animals were weighed, vaccinated against clostridial disease, dewormed, supplemented with an oral vitamin and mineral supplement, and labelled. The animals were confined in separate pens of 1 × 2 m, equipped with a trough and a drinking fountain, in an open shed. The experimental period was 84 days, with an adaptation period of 10 days.

The diets used were based on Tifton hay, forage cactus silage, corn meal, soybean meal, wheat bran, and a mineral supplement to meet the requirements for a weight gain of 150 g/day, according to the NRC [7] (Tables 1 and 2).

**Table 1:** Chemical composition (g/kg of DM) of the ingredients.

Items	Ground corn	Soybean meal	Wheat bran	Tifton hay	Forage cactus silage
**Dry matter**	872.00	886.00	866.70	887.50	73.90
**Organic matter**	980.00	928.70	949.50	937.30	830.80
**Ash**	20.00	71.30	50.50	62.70	169.20
**Ether extract**	48.40	3.60	24.70	16.40	26.40
**Crude protein**	104.40	529.50	198.30	65.80	83.10
**aNDFom**^**+**^	388.20	213.40	419.60	614.10	428.40
**ADF**^**¶**^	34.50	128.00	125.20	403.60	279.10
**TC**^**†**^	827.20	395.50	726.50	865.00	721.30
NFC*	438.90	182.10	306.90	250.90	292.80
**Cellulose**	25.50	123.80	85.50	340.50	224.00
**Hemicellulose**	353.70	85.30	294.40	210.50	149.30
**ADL**^**♦**^	8.90	4.20	39.70	63.10	55.10

^+^aNDFom, neutral detergent fibre assayed with a heat stable amylase and expressed exclusive of residual ash

^¶^ADF, acid detergent fibre

^†^TC, total carbohydrates

*NFC = non-fibre carbohydrates

^♦^ADL = acid detergent lignin.

**Table 2:** Composition of ingredients and chemical composition of the experimental diets.

Ingredient (g/kg DM)	Forage cactus silage (% DM)		
	0	21	42
**Ground corn**	280.00	230.00	180.00
**Soybean meal**	80.00	100.00	120.00
**Wheat bran**	30.00	60.00	90.00
**Tifton hay**	600.00	390.00	180.00
**Forage cactus silage**	0.00	210.00	420.00
**Mineral supplement**	10.00	10.00	10.00
**Chemical composition (g/kg)**			
**Dry matter**	858.20	697.50	536.80
**Organic matter**	936.00	915.60	895.30
**Crude protein**	129.30	130.05	139.20
**Ether extract**	29.40	28.20	27.10
**aNDFom**^**+**^	491.10	445.10	399.20
**Acid detergent fibre**	259.90	236.80	213.70
**Ash**	64.00	84.40	104.70
**Acid detergent lignin**	39.80	38.70	37.50
**Hemicellulose**	231.20	208.30	185.50
**Cellulose**	220.10	198.10	176.10
**Total carbohydrates**	787.90	755.90	724.00
**Non-fibre carbohydrates**	296.80	310.80	324.80
**TDN**^**¶**^	660.00	661.30	663.50
**Dry matter Digestibility (g/kg)**	632.52	666.51	681.60

^+^aNDFom, neutral detergent fibre assayed with a heat stable amylase and expressed exclusive of residual ash

^¶^TDN, total digestible nutrients was calculated according to Weiss [14].

The roughage:concentrate ratio was 60:40, and forage cactus silage (*Opuntia fícus-indica* Mill) was used to replace three proportions of hay in the diet (0, 21, and 42% of the DM). The diets were supplied *ad libitum* twice a day at 9 a.m. and 3 p.m. Feed refusals were weighed daily to determine feed intake, and the quantity offered was calculated as a function of the previous day’s supply based on 10% refusals. Water was supplied intermittently according to the respective treatments. In the treatment 0 h, the animals received water daily; in the 24-h treatment, they received water for only 1 day and were then without water for 24 h. In the 48-h treatment, the animals received water for 1 day and were then without water for 48 h. In the days of water supply, water was provided *ad libitum* in the morning.

The forage cactus crop for silage was harvested after 2 years of regrowth; all cladodes, except the main and the primary ones, were harvested, processed in a stationary forage machine to achieve a particle size 2 × 2 cm, and ensiled in drums with a capacity of 200 L. The silos were opened 60 days after ensiling when the experiment was started. Silage samples were collected at the time of supplying the diets every 15 days for the analysis of pH and ammoniacal nitrogen [8], and organic acids [9]. The silage had the following fermentative characteristics: pH 4.02, lactic acid content 60.0 g/kg DM, acetic acid content 1.5 g/kg DM, butyric acid content 0.15 g/kg DM, and ammoniacal nitrogen content 1.05% total nitrogen.

The chemical composition of the ingredients and of the diets in dry matter (DM), organic matter (OM), ash, ether extract (EE), cellulose (CEL), acid detergent lignin (ADL), hemicellulose (HEM) and the crude protein (CP) by the Kjeldahl method were analysed by the AOAC [10] methodology. The neutral detergent fibre (aNDFom) content using heat stable amylase and sodium sulphite and expressed without residual ash and acid detergent fibre (ADF) [11].

Equations were used to estimate the total carbohydrate levels (TC) [12], the non-fibrous carbohydrate (NFC) level [13] and TDN [14].

The DM intake, DM intake of the lambs (kg/animal/day), Feed conversion (feed:gain), average daily gain (ADG), were estimated from feed intake, final and initial body weights and the number of days in the experimental period (84 days). The animals were weighed after being subjected to a solid feed fasting period of 16 h.

#### Slaughtering and assessment procedures

After 84 days of the experiment, the animals were subjected to solid fasting for 16 h and subsequently weighed to obtain the live weight at slaughter (LWS).

The animals were slaughtered by concussion stunning using a captive bolt pistol (Model Tec 10 P, Ctrade®, Porto Alegre, RS, Brazil) followed by bleeding for 3 min by carotid and jugular section. The animals were then skinned and eviscerated to obtain the edible parts which are not constituents of the carcass (blood, tongue, lungs, heart, liver, kidney, spleen, gastrointestinal tract (GIT), and omentum).

Afterwards, the head, feet, and tail were removed, and the hot carcass weight (HCW) was determined immediately. The empty body weight (EBW) was determined by the difference between live weight at slaughter and the weight of the gastrointestinal tract content.

The carcass pH was determined by measuring the *Longissimus lumborum* muscle 0 and 24 h after slaughter, using a portable pH meter (model Testo 205, Campinas, SP, Brazil) previously calibrated with pH 4.0 and 7.0 buffer solutions.

The carcasses were cooled for 24 h at 4°C in a cold store. At the end of this period, the cold carcass weight (CCW) and loss by cooling were obtained. Subsequently, the hot and cold carcass yields were obtained using the equations.

Morphometric measurements were made on the carcasses and later on the half carcasses which was determined the carcass compactness index (CCI), after the morphometric measurements the half carcasses were then weighed and sectioned into five anatomical regions (commercial cuts): chuck, leg, loin, rib, and neck [15, 16].

In the left half-carcass, a cross section was also performed between the 12th and 13th ribs to measure the rib eye area (REA) of the *Longissimus dorsi* muscle.

The left legs and loins were labelled, packed, and frozen at -18°C for further analyses.

#### Dissection of the legs

The leg dissection procedures were performed according to the method described by Brown and Williams [17]. The legs were removed from the freezer 24 h prior to the dissection and thawed in a refrigerator at a temperature of approximately 10°C. The other tissues (veins, arteries, tendons, and lymph nodes) were weighed.

The leg muscle ratio was determined according to Purchas *et al*. [18], using the weights of the five femoral muscles (W5M) (M. *Biceps femoris*, M. *Semimembranosus*, M. *Semitendinosus*, M. *Adductor femoris*, and M. *Quadriceps femoris*) and the leg muscle index (LMI) was calculated by the equation = $\frac{\sqrt{W5M/FL}}{FL}$.

#### Physicochemical characteristics and approximate composition

Physical analyses of the muscle *Longissimus lumborum* (pH, colour, cooking losses, and shear force) were performed.

For this, the left loins were thawed in plastic bags in a refrigerator at 10°C and subsequently dissected to obtain the *Longissimus lumborum* muscle; all fat was removed from the sample.

Two steaks with a thickness of 2.5 cm were exposed to the atmosphere for 50 min to determine the colour indices L*, a*, and b* (luminosity, redness, and yellowness, respectively) with a colorimeter (MINOLTA model CR-400, Osaka, Japan), calibrated to manufacturer's recommendation; three readings were performed at different locations on the muscle (*Longissimus lumborum*) [19]. The saturation index (Chroma) was determined according to the equation: $Chroma=\sqrt{a^{2}+b^{2}}$.

To determine the cooking losses of the *Longissimus lumborum*, the steaks were thawed in a refrigerator for 24 h, weighed precision scale (model TX3202L, Shimadzu, Campinas, SP, Brazil). Afterwards, they were baked in a preheated electric oven (model Star, Fischer, Campinas, SP, Brazil) until the internal temperature of the samples reached 71°C using a digital reading (TM-361, Tenmars Electronics, Taiwan, China). The samples were then weighed to obtain the weight lost by the AMSA [20] methodology.

For the shear force analysis, the samples used for the determination of cooking losses were cooled in a refrigerator at 4°C for 24 h. After this period, at least three cores of 1.27 cm in diameter and 2.0 cm in length that were parallel to the muscle fibres were removed from each sample using a cork borer. The shear force was measured via a texturometer equipped with a Warner-Bratzler stainless-steel blade (model 3000, G-R Manufacturing, Kansas, USA) by the AMSA [20] methodology.

The moisture, ash, and protein content were evaluated by the AOAC [10] methodology and the ether extract (EE) content was determined by the AOCS [21] methodology.

#### Fatty acid profile and nutraceutical parameters

To analyse the fatty acid (FA) profile, the samples of *Longissimus lumborum* muscles without epimysium and the subcutaneous were thawed and macerated and homogenised in a micro propeller turbine (Turratec Tecnal, Piracicaba, SP, Brazil). For lipid extraction, the method described by Hara and Hadin [22], using an n-hexane-isopropanol solution (3:2 v/v), was used. After extraction, the lipids were esterified and methylated [23]. The transmethylated samples were analysed in a gas chromatograph (Focus GC AI 3000, Thermo Finnigan analyser, Milan, Italy) with a flame ionisation detector and a CP-Sil 88 (Varian) capillary column, 100 m long by 0.25 μm internal diameter and 0.20 μm film thickness. Hydrogen was used as the carrier gas at a flow rate of 1.8 mL/min. The initial oven temperature programme was 70°C, waiting time 4 min, 175 ºC (13 ºC/min), waiting time 27 min, 215 ºC (40 ºC/min), waiting time 9 min, with an increase by 7°C/min up to 230 ºC, remaining for 5 min, totalling 65 min. The steamer temperature was 250°C and the detector temperature was 300°C.

A 1-μL aliquot of the esterified extract was injected into the chromatograph and FA identification was performed by comparing the retention times. The percentages of the fatty acids were obtained using the software Chromquest 4.1 (Thermo Electron, Milan, Italy).

The fatty acids were identified by comparing the retention times of the methyl esters of the samples with butter fatty acid standards (BCR-CRM 164, Anhydrous Milk-Fat Producer: BCR Institute for Reference Materials and Measurements; Supelco TM Component FAME Mix, cat 18919 Supelco, Bellefonte, PA). To quantify the fatty acid methyl esters, a response factor was generated for each fatty acid based on the standard sample. The results were quantified by normalising the areas of the methyl esters and expressed as an area percentage (%).

After identification of the fatty acids, the total saturated fatty acids (SFA), monounsaturated fatty acids (MUFA), and polyunsaturated fatty acids (PUFA), and MUFA:SFA, PUFA:SFA, PUFA:MUFA, and n6:n3 ratios were calculated.

The nutritional quality of the lipid fraction was evaluated through the atherogenicity index (AI) using the equation AI = [(C_12:0_ + (4 × C_14:0_) + C_16:0_)] / (∑MUFA + C_18:1 cis9_ + Σn6 + Σn3), the thrombogenicity index (TI) using the fatty acids ratio TI = [(C_14:0_ + C_16:0_ + C_18:0_) / [(0.5 × C_18:1 cis9_) + ((0.5 × ΣMUFA) + (0.5 × Σ n6) + (3 × Σ n3) + (Σn3 / Σn6)] according to Ulbricht and Southgate [24]; the hypocholesterolemic and hypercholesterolemic (h:H) fatty acids ratio by [25] equation h:H = (C_18:1 cis9_ + C_18:2 n6_ + C_20:4 n6_ + C_18:3 n3_ + C_20:5 n3_ + C_22:5 n3_ + C_22:6 n3_) / (C_14:0_ + C_16:0_); and the desirable fatty acids (DFA) using the [26] equation DFA = (ΣMUFA + ΣPUFA + C_18:0_).

The activities of Δ9-desaturase C16, Δ9-desaturase C18, and elongase were estimated by [27] equations: Δ9-desaturase C16 = [C_16:1_ / (C_16:0_ + C_16:1_)] × 100; Δ9-desaturase C18 = [(C_18:1 cis–9_) / (C_18:0_ + C_18:1 cis–9_)] × 100; and elongase = [(C18:0 + C18:1 cis–9) / (C16:0 + C16:1 + C18:0 + C18:1 cis–9)] × 100.

#### Experimental design and statistical analyses

The experimental design consisted of randomised blocks, distributed according to the animals’ weights. The treatments were arranged in a 3 × 3 factorial design comprising three addition ratios of cactus silage in the diet (0, 21, and 42% based on DM) and three water supply intervals (0, 24, and 48 h), with four replicates. The following mathematical model was used:

AYijk=μ+αi+βj+αβij+k+eijk

where Y is the observed value of the variable ijk that refers to the k-th repetition of the combination of the i-th level of factor A with the j-th level of factor B, μ is the average of all experimental units for the variable, αi is the effect of the ratios of forage cactus silage (i = 0, 21, and 42%) at the observed value Yijk, βj is the effect of the intermittent water supply (j = 0, 24, and 48 h) at the observed value Yijk, αβij is the effect of the interaction between the ratio of forage cactus silage and intermittent water supply, k is the block effect on the observation Yijk, and eijk is the error associated with the observation of Yijk.

The data were subjected to analysis of variance (ANOVA) using the Proc GLM of SAS 9.2 [28]. Tukey’s test was applied at a probability level of 5% to compare the treatment averages.

## Results

### Morphometric measurements

There was no significant effect of intermittent water supply nor a significant effect of the interaction between water supply and cactus silage (*P* > 0.05) on the morphometric measurements or carcass compactness index (CCI) (Table 3). In contrast, the morphometric measurements and CCI were affected by the diet (*P* < 0.05), except for croup width. Animals that did not receive cactus silage as a substitute for Tifton hay presented lower values, showing less muscle development.

**Table 3:** Morphometric measurements of lamb carcasses.

Item	Forage cactus silage (% DM)			Water supply (h)			SEM	P-value^+^		
	0	21	42	0	24	48		FC	W	FC × W
**Internal length (cm)**	57.78^b^	61.62^a^	59.79^ab^	59.72	59.39	60.08	0.997	0.037	0.887	0.414
**Leg length (cm)**	37.41^b^	40.33^a^	40.12^a^	39.16	39.79	38.91	0.688	0.010	0.662	0.707
**Thoracic width (cm)**	14.95^b^	15.65^ab^	16.71^a^	15.39	15.65	16.28	0.364	0.007	0.224	0.879
**Croup width (cm)**	20.08	20.21	21.95	20.12	20.56	21.23	0.524	0.072	0.076	0.673
**Thoracic perimeter (cm)**	63.36^b^	68.31^a^	69.91^a^	67.41	66.45	67.73	0.841	<0.001	0.539	0.994
**Croup perimeter (cm)**	57.65^b^	59.69^ab^	61.17^a^	59.14	58.85	60.52	0.894	0.032	0.380	0.867
**Leg perimeter (cm)**	28.48^b^	31.78^a^	31.20^ab^	29.70	30.05	31.71	0.850	0.023	0.220	0.809
**Thorax depth (cm)**	15.72	16.50	17.06	16.10	16.41	16.77	0.370	0.052	0.445	0.476
**Carcass compactness index (kg/cm)**	0.20^b^	0.25^a^	0.27^a^	0.24	0.23	0.24	0.010	<0.001	0.639	0.944

The values within a row with different superscripts are significantly different.

^+^FC, forage cactus silage; W, water supply; FC × W, interaction between forage cactus silage and water supply.

Lambs fed with 21% cactus silage as a substitute for Tifton hay presented higher internal carcass length (*P* = 0.037) and leg perimeter (*P* = 0.023) values than animals fed only with Tifton hay. The thorax width (*P* = 0.007) and croup perimeter (*P* = 0.032) were higher (16.71 and 61.17 cm, respectively) in lambs with the addition of 42% cactus silage in the diet than those that only received Tifton hay as roughage. Animals fed with diets containing forage cactus silage showed values higher (*P* < 0.001) to carcass compactness index (CCI).

### Non-carcass constituents and carcass traits

There was no significant effect of the intermittent water supply (*P* > 0.05) or the interaction between the water supply and cactus silage (*P* > 0.05) on the non-carcass constituents, except for the spleen, which presented a significant difference (*P* = 0.04) between 24 and 48 hours of intermittent water supply with averages of 0.05 and 0.08 kg, respectively.

There was a significant effect (*P* < 0.05) on weight of the blood (*P* = 0.007), liver (*P* = 0.001), perirenal fat (*P* = 0.002), and empty gastrointestinal tract (GIT, *P* = 0.022) of the addition of forage cactus silage, with the highest averages for the treatment with 42% forage cactus silage. The weight of blood varied from 0.72 to 0.78 kg, liver from 0.38 to 0.54 kg, perirenal fat 0.19 to 0.32, and empty gastrointestinal tract from 2.48 to 2.98 kg for the treatments without silage and with 42% forage cactus silage.

The other organs did not differ significantly between the treatments (*P* > 0.05).

There was no significant effect of the interaction between water supply and cactus silage (*P* > 0.05) on the carcass traits. There was no effect of intermittent water supply (*P* > 0.05) up to 48 h on the DM intake or carcass traits (Table 4). However, feed conversion (*P* = 0.007) and the average daily gain (*P* = 0.007) were affected by the intermittent water supply.

**Table 4:** Dry matter and water intake, hydric balance, feed conversion and carcass traits of lambs.

Item	Forage cactus silage (% DM)			Water supply (h)			SEM	P-value^+^		
	0	21	42	0	24	48		FC	W	FC × W
**Water Intake (kg)**	1.43^c^	2.52^b^	3.81^a^	2.81	2.38	2.53	0.19	<0.001	0.093	0.801
**HB (g)**	937.00^b^	1666.50^a^	1852.10^a^	1523.90	1663.50	1328.80	96.33	< .0001	0.224	0.767
**Dry matter intake (g/day)**	657.22^b^	795.11^a^	839.46^a^	791.55	724.77	775.47	25.51	0.002	0.331	0.968
**Feed conversion**	5.00^a^	4.37^ab^	4.11^b^	4.70^a^	4.90^a^	3.86^b^	0.49	0.028	0.007	0.081
**Average daily gain (g/day)**	142.00^b^	184.00^a^	208.00^a^	173.42^ab^	159.12^b^	200.78^a^	24.75	0.028	0.007	0.081
**Slaughter weight (kg)**	28.39^b^	32.91^ab^	33.41^a^	31.60	30.55	32.56	1.328	0.023	0.571	0.982
**Empty body weight (kg)**	22.27^b^	27.86^a^	28.88^a^	26.27	25.41	27.32	1.159	0.001	0.507	0.995
**Hot carcass weight (kg)**	12.25^b^	15.74^a^	16.45^a^	14.79	14.36	15.28	0.698	0.001	0.653	0.947
**Cold carcass weight (kg)**	11.97^b^	15.42^a^	16.14^a^	14.49	14.07	14.97	0.687	0.001	0.658	0.944
**Cooling losses (%)**	2.27^a^	2.01^b^	1.91^b^	2.07	2.05	2.08	0.072	0.035	0.977	0.347
**Hot carcass yield (%)**	43.23^b^	47.68^a^	49.19^a^	46.58	46.77	46.74	0.669	0.001	0.978	0.116
**Cold carcass yield (%)**	42.24^b^	46.71^a^	48.25^a^	45.62	45.81	45.78	0.665	0.001	0.977	0.113
**Biological yield (%)**	55.20	56.35	56.94	56.11	56.49	55.88	0.891	0.387	0.886	0.068
**Ribeye area (cm**^**2**^**)**	11.38^b^	11.20^b^	13.98^a^	12.40	11.70	12.46	0.428	0.001	0.386	0.277

The values within a row with different superscripts are significantly different.

^+^FC, forage cactus silage; W, water supply; FC × W, interaction between forage cactus silage and water supply

^¶^(kg of feed/kg BW gain)

The addition of forage cactus silage as a substitute for Tifton hay promoted an increase in the average daily gain (*P* = 0.028) and water (*P* < 0.001) and DM (*P* = 0.002) intake, which might explain its effect on the carcass traits in this study. In addition, a better feed conversion (*P* < 0.001) was found for animals fed diets containing forage cactus silage. The addition of forage cactus silage as a substitute for Tifton hay also influenced the slaughter weight (*P* = 0.023) and empty body weight (*P* = 0.001), and hence the carcass weights (*P* = 0.001). However, the biological yield (BY) was not affected (*P* = 0.387) by forage cactus silage addition. The average hot and cold carcass weights were higher for animals fed with cactus silage, probably due to the higher weight at slaughter achieved with this diet. The animals fed with cactus silage showed reduced (P < 0.035) losses by cooling. The addition of 42% cactus silage to the diet increased (*P* = 0.001) the rib eye area (13.98 cm^2^).

There was no significant effect (*P* = 0.322) of water restriction (*P* = 0.098) or the interaction between water supply and cactus silage on the weights of commercial cuts (Table 5). The addition of cactus silage as a substitute for Tifton hay positively affected (*P* = 0.001) the commercial cuts carcass weight of the lambs.

**Table 5:** Cold carcass and commercial cuts weights of lambs.

Item	Forage cactus silage (% DM)			Water supply (h)			SEM	P-value^+^		
	0	21	42	0	24	48		FC	W	FC × W
**Cold half-carcass**	5.85^b^	7.61^a^	8.28^a^	6.98	7.16	7.32	0.153	0.001	0.733	0.427
**Weight cuts (kg)**										
**Shoulder**	1.16^b^	1.48^a^	1.56^a^	1.36	1.39	1.40	0.058	0.001	0.882	0.462
**Neck**	0.65^b^	0.80^ab^	0.95^a^	0.80	0.79	0.78	0.046	0.001	0.963	0.645
**Ribs**	1.48^b^	1.98^a^	2.21^a^	1.81	1.82	1.97	0.086	0.001	0.322	0.098
**Loin**	0.69^b^	0.95^a^	1.02^a^	0.85	0.90	0.87	0.055	0.001	0.758	0.431
**Leg**	1.84^b^	2.40^a^	2.48^a^	2.18	2.22	2.24	0.108	0.001	0.910	0.661

The values within a row with different superscripts are significantly different.

^+^FC, forage cactus silage; W, water supply; FC × W, interaction between forage cactus silage and water supply.

Among the commercial cuts weights of the half carcass, only the rib yield was significantly influenced (*P* = 0.026) by the interaction between the addition of cactus silage and the effect of intermittent water supply (Table 6). The animals fed a diet without the addition of cactus silage and with an intermittent water supply of 48 h showed lower rib yields (24.36%) than animals from the diets without the addition of cactus silage (28.46%).

**Table 6:** Rib yield of carcasses of lambs.

Item	Forage cactus silage (% DM)	Water supply (h)		
		0	24	48
	0	28.46^Aa^	24.73^ABa^	24.36^Bb^
**Ribs (%)**	21	24.64^Aa^	25.46^Aa^	27.97^ABa^
	42	25.94^Aa^	25.97^Aa^	28.84^Aa^

The values within a row with uppercase and within a column with lowercase superscripts are significantly different

There was no effect of intermittent water supply up to 48 h on the tissue composition and leg muscle index (Table 7). The addition of forage cactus silage increased the weight of the leg (*P* = 0.005), muscle (*P* = 0.003), fat (*P* = 0.019), and bone (*P* = 0.004).

**Table 7:** Tissue composition and leg muscle index of lambs.

Item	Forage cactus silage (% DM)			Water supply (h)			SEM	P-value^+^		
	0	21	42	0	24	48		FC	W	FC × W
**Leg (kg)**	1.82^b^	2.39^a^	2.38^a^	2.15	2.22	2.22	0.121	0.005	0.893	0.852
**Muscle (kg)**	1.16^b^	1.51^a^	1.58^a^	1.36	1.45	1.41	0.078	0.003	0.707	0.642
**Fat (kg)**	0.20^b^	0.28^ab^	0.33^a^	0.28	0.29	0.24	0.029	0.019	0.490	0.196
**Bone (kg)**	0.29^b^	0.38^a^	0.38^a^	0.33	0.35	0.36	0.019	0.004	0.583	0.646
**Others tissue (kg)**	0.07^a^	0.13^b^	0.10^ab^	0.09	0.09	0.13	0.013	0.031	0.057	0.116
**Muscle:Bone**	3.99	3.95	4.10	4.12	4.05	3.87	0.201	0.866	0.663	0.706
**Muscle:Fat**	5.98	5.67	5.03	5.12	5.62	5.94	0.547	0.474	0.576	0.103
**Leg muscle index**	0.38	0.39	0.40	0.38	0.40	0.40	0.012	0.240	0.421	0.172

The values within a row with different superscripts are significantly different

^+^FC, forage cactus silage; W, water supply; FC × W, interaction between forage cactus silage and water supply.

### Physicochemical characteristics and approximate composition

There was no effect of intermittent water supply (*P* = 0.209) up to 48 h on the physical and chemical parameters of the *Longissimus lumborum* muscle (Table 8). Lower levels of L* (*P* = 0.020), a* (*P* = 0.016), b* (*P* = 0.023), and Chroma (*P* = 0.003) were observed in the *Longissimus lumborum* muscle of lambs derived from treatments with a higher addition level of cactus silage in the diet than those from the treatment without cactus silage.

**Table 8:** Physicochemical characteristics and proximate composition of the *Longissimus lumborum* of lambs.

Item	Forage cactus silage (% DM)			Water supply (h)			SEM	P-value^+^		
	0	21	42	0	24	48		FC	W	FC × W
**pH**_**0h**_	6.44	6.39	6.26	6.37	6.38	6.34	0.059	0.096	0.884	0.376
**pH**_**24h**_	5.70	5.62	5.74	5.75	5.68	5.63	0.075	0.547	0.481	0.505
Lightness (L*)	40.56^a^	37.83^ab^	36.97^b^	39.33	38.15	37.89	0.847	0.020	0.455	0.142
Redness (a*)	17.94^a^	16.30^ab^	15.61^b^	16.51	17.47	15.87	0.525	0.016	0.124	0.770
Yellowness (b*)	10.60^a^	10.34^ab^	9.05^b^	10.30	10.26	9.42	0.384	0.023	0.211	0.091
**Chroma**	20.84^a^	19.30^ab^	18.04^b^	19.46	20.26	18.46	0.476	0.003	0.051	0.813
**Cooking losses (%)**	35.56	35.08	33.93	36.28	34.59	33.70	1.332	0.676	0.386	0.591
**Shear force (kg/cm**^**2**^**)**	1.81	1.63	1.52	1.57	1.66	1.72	0.103	0.165	0.601	0.270
**Approximate composition (%)**										
**Moisture**	73.05	73.94	73.81	73.66	73.67	73.48	1.986	0.157	0.909	0.708
**Protein**	22.59	21.59	21.90	22.22	21.73	22.14	0.763	0.070	0.455	0.781
**Ash**	1.03	1.02	1.03	1.04	1.03	1.02	0.065	0.586	0.209	0.297
**Ether extract**	2.06	2.08	2.09	2.04	2.08	2.10	0.098	0.882	0.573	0.954

The values within a row with different superscripts are significantly different

^+^FC, forage cactus silage; W, water supply; FC × W, interaction between forage cactus silage and water supply.

An intermittent water supply of up to 48 h with the addition of cactus silage in the diet did not affect (*P* = 0.091) the approximate composition of the lambs’ meat.

### Fatty acid profile of meat

In relation to the fatty acid profile of the meat, there was no significant effect of the intermittent water supply (*P* = 0.141) or interaction between the water supply and cactus silage (*P* = 0.074). The intermittent water supply did not affect (*P* = 0.110) the levels of saturated fatty acids (SFA), monounsaturated fatty acids (MUFA), or polyunsaturated fatty acids (Table 9). The addition of forage cactus silage to the diets of lambs affected (*P* = 0.044) the composition of some SFA, MUFA, and PUFA in the meat.

**Table 9:** Fatty acid composition in the *Longissimus lumborum* muscle of lambs.

Item (%)	Forage cactus silage (% DM)			Water supply (h)			SEM	P-value^+^		
	0	21	42	0	24	48		FC	W	FC × W
**C12:0**	0.06	0.06	0.07	0.06	0.06	0.07	0.007	0.694	0.723	0.592
**C14:0**	1.73	1.83	2.00	1.75	1.82	1.96	0.112	0.279	0.440	0.802
**C14:0**_**iso**_	0.04^a^	0.02^ab^	0.02^b^	0.02	0.03	0.04	0.005	0.023	0.278	0.483
**C15:0**	0.27	0.24	0.23	0.24	0.24	0.27	0.017	0.334	0.245	0.413
**C15:0**_**iso**_	0.14^a^	0.10^b^	0.09^b^	0.10	0.12	0.12	0.008	0.001	0.185	0.616
**C15:0**_**anteiso**_	0.17^a^	0.11^b^	0.10^b^	0.11	0.13	0.14	0.013	0.002	0.278	0.876
**C16:0**	24.11	25.53	25.21	24.74	24.46	25.56	0.612	0.242	0.434	0.743
**C16:0**_**iso**_	0.20^a^	0.15^ab^	0.12^b^	0.13	0.16	0.18	0.018	0.017	0.226	0.211
**C17:0**	1.00	1.01	1.52	1.43	1.61	1.30	0.041	0.774	0.351	0.579
**C18:0**	17.56^a^	17.08^ab^	15.26^b^	16.74	16.98	16.40	0.626	0.048	0.800	0.225
**C21:0**	0.11^a^	0.07^ab^	0.05^b^	0.08	0.08	0.06	0.001	0.007	0.225	0.456
**Other SFA**^**♦**^	0.63	0.62	1.18	1.23	1.16	1.25	0.029	0.541	0.831	0.894
**C14:1**_**cis9**_	0.06^b^	0.07^ab^	0.08^a^	0.07	0.06	0.07	0.006	0.044	0.776	0.971
**C16:1**_**cis9**_	2.12	2.19	2.32	2.18	2.13	2.30	0.083	0.252	0.316	0.380
**C18:1**_**cis9**_	38.54	38.11	39.77	38.38	39.08	38.75	0.802	0.359	0.829	0.253
**C18:1 cis11**	3.58	3.94	3.83	3.77	3.81	3.74	0.199	0.417	0.967	0.560
**C20:1**	0.15^a^	0.10^b^	0.12^a^	0.12	0.13	0.12	0.007	0.001	0.503	0.074
**Other MUFA**^**ᴥ**^	4.95	4.71	4.72	5.02	4.75	4.69	0.212	0.530	0.471	0.616
**C18:2**_**cis-9 cis-12**_	2.40	2.18	2.54	2.59	2.38	2.15	0.268	0.646	0.531	0.628
**C18:2**_**cis-9 trans-11**_	0.21	0.21	0.21	0.23	0.20	0.21	0.026	0.996	0.674	0.569
**C18:3 n6**	0.02	0.02	0.02	0.02	0.02	0.02	0.003	0.477	0.688	0.705
**C18:3 n3**	0.17^a^	0.11^b^	0.11^b^	0.13	0.15	0.12	0.014	0.010	0.405	0.396
**C20:4 n6**	1.03	1.00	1.01	1.25	1.00	0.83	0.138	0.986	0.141	0.792
**C20:5 n3**	0.19	0.10	0.12	0.14	0.17	0.10	0.035	0.144	0.464	0.612
**Other PUFA**^**¶**^	0.36	0.22	0.23	0.27	0.31	0.22	0.041	0.110	0.531	0.619

The values within a row with different superscripts are significantly different

^+^FC, forage cactus silage; W, water supply; FC × W, interaction between forage cactus silage and water supply.

^**♦**^SFA, saturated fatty acids

^ᴥ^MUFA, monounsaturated fatty acids

^¶^PUFA, polyunsaturated fatty acids.

The intermittent water supply did not affect (P = 0.187) the group sums and ratios for FA, nor the nutraceutical parameters. The addition of forage cactus silage to the diet of lambs affected (P = 0.029) the sum n-3 only, with the highest value observed in the treatment without forage cactus silage, however, this did not differ statistically from the sum n-3 found in the meat of lambs fed with 42% cactus silage (Table 10).

**Table 10:** Group sums and ratios of FA and nutraceutical parameters of the *Longissimus lumborum* muscle of lambs.

Item (%)	Forage cactus silage (% DM)			Water supply (h)			SEM	P-value^+^		
	0	21	42	0	24	48		FC	W	FC × W
**Group sums and ratios**										
**ΣSFA**^**♦**^	46.02	46.82	45.85	46.63	46.85	47.35	0.977	0.352	0.774	0.294
**ΣMUFA**^**ᴥ**^	49.40	49.12	50.84	49.54	49.96	49.67	0.916	0.408	0.947	0.206
**ΣPUFA**^¶^	4.38	3.84	4.24	4.63	4.23	3.65	0.459	0.682	0.354	0.733
**ΣPUFA:ΣSFA**	0.10	0.08	0.09	0.10	0.09	0.08	0.010	0.510	0.374	0.780
**ΣPUFA:ΣMUFA**	0.09	0.08	0.08	0.09	0.08	0.07	0.009	0.635	0.424	0.735
**ΣMUFA:ΣSFA**	1.07	1.05	1.11	1.06	1.07	1.05	0.040	0.450	0.871	0.212
**Σn-6**	3.7	3.44	3.81	4.12	3.63	3.24	0.139	0.918	0.189	0.827
**Σn-3**	0.68^a^	0.39^b^	0.43^ab^	0.51	0.6	0.41	0.041	0.029	0.387	0.502
**n-6:n-3**	5.44	8.60	8.86	8.08	6.05	7.90	0.645	0.099	0.291	0.117
**Nutraceutical parameters**										
**Desirable fatty acids**	71.34	70.04	70.34	70.91	71.17	69.72	0.659	0.257	0.204	0.702
**Atherogenicity index**	0.34	0.36	0.35	0.34	0.34	0.36	0.027	0.402	0.362	0.519
**Thrombogenicity index**	0.90	0.95	0.87	0.91	0.90	0.93	0.069	0.398	0.746	0.242
**h:H index**	1.66	1.53	1.62	1.62	1.65	1.54	0.059	0.221	0.375	0.551
**Δ 9-desaturase C16**	8.08	7.90	8.43	8.10	8.01	8.26	0.300	0.564	0.779	0.471
**Δ 9-desaturase C18**	68.70	69.05	72.27	69.63	69.71	70.26	1.064	0.126	0.871	0.209
**Elongase**	68.14	66.57	66.65	67.19	67.83	66.44	0.636	0.132	0.187	0.681

The values within a row with different superscripts are significantly different

^+^FC, forage cactus silage; W, water supply; FC × W, interaction between forage cactus silage and water supply.

^**♦**^SFA, saturated fatty acids total

^ᴥ^MUFA, monounsaturated fatty acids total

^¶^PUFA, polyunsaturated fatty acids total.

## Discussion

The direct result of water restriction is a reduction in feed intake and, hence, reduced body weight [4]. However, the inclusion of cactus silage promoted a higher intake of water, an increase in dry matter intake, and a reduction in the loss of body water; the animals had a performance close to the 150 g average daily weight gain aimed for in the diet formulation, indicating that the adaptations of the lambs were sufficient to overcome the water deficit. Adapted sheep deal with water restriction via physiological adjustments such as reduced water loss from the rumen by reducing its volume, reduced body weight, and an increased extraction of water from faeces and urine [29].

The inclusion of cactus levels favored the animals' hydric balance, which may have a beneficial effect in preventing the presence of urolithiasis, according to Alhanafi et al. [30] claiming that diets containing cactus although not toxic to ruminants, have a high concentration of calcium oxalate tending to cause lithiases and reduce animal performance which was not observed.

Forage cactus silage shows a proper fermentation profile, with almost adequate levels of lactic acid and concentrations of acetic, propionic, and butyric acid [6], which suggests higher DM intake levels, explaining the higher average daily gain and the positive effects on carcass characteristics. In addition, forage cactus has high acceptability, which makes it generally consumed voluntarily in large quantities [29, 31].

Besides silage intake, its digestibility has a great effect on weight gain. Organic matter in forage cactus is highly degradable [32], promoting a higher nutrient intake through diet and hence a higher availability of nutrients for tissue synthesis, which could explain the higher weight gain observed in our study.

Dry matter digestibility was not affected by cactus silage inclusion, however, the lower amount of aNDFom and ADF increased the passage rate of cactus diets and thus the animals had increased dry matter intake. Even though there was no difference in digestibility, the animals had higher nutrient intake and thus higher performance and lower feed conversion. Higher DM intake have also been observed by Costa et al. [31], who used cactus as a substitute for corn in the diets of sheep.

Higher weight at slaughter was observed for animals who received higher cactus silage levels. The higher weight is related to diet intake and digestibility. According to Barroso et al. [33], the high digestibility coefficient of the cactus is mainly due to pectin, resulting in a better animal performance. The higher intake of the animals with 42% silage in the diet probably promoted more tissue development, resulting in heavier and larger animals.

The absence of any effects of intermittent water supply on carcass morphometric characteristics suggests that the intermittent water supply in the evaluated forms was not detrimental to the animals and did not affect muscle and bone development. It also showed the ability of sheep to tolerate up to 2 days with water restriction did not compromise morphometric characteristics. This result is significant for the raising of sheep in arid and semiarid regions.

Probably the explanation of water restriction tolerance is related to the storage of endogenous water in the form of branched fat at strategic points in the body, just as camelids that store branched fat in large quantities in the hump. Sheep of semiarid breed such as the Damara that store branched fat in the fat tail.

According to Casamassima et al. [34], the ability to respond to water restriction depends on the breed. However, mechanisms to overcome water deficits are generally more efficient in the short term, with reduced efficiency over longer periods. Chedid et al. [4] observed that indigenous sheep from semiarid and arid conditions could withstand more than 1 month without significant physiological changes by ingesting water only every 2 days, whereas access to water every 5 days resulted in relevant changes. Similarly, Al-Ramamneh et al. [5] found that water restriction of up to 2 days in lambs and goats did not induce significant changes in body mass.

The increase in the morphometric characteristics values of lambs fed with diets containing 42% forage cactus silage reflects the increase in carcass weight, with a higher amount and distribution of muscle mass over the bone base [35]. Since forage cactus supplies energy readily available in the rumen, favouring the synthesis of microbial protein and the production of volatile fatty acids, it also promotes higher feed use by the animal and hence more growth and muscle development, which is reflected in more carcass elongation and muscle deposition. The highest CCI was expected for the animals fed with forage cactus silage, since the relationship between cold carcass weight and internal carcass length was used to determine CCI, which is affected by cactus silage. The diets containing 21 and 42% cactus silage showed higher contents of CP (13.50 and 14.32%, respectively) than that without silage (11.77%), as well as higher NFC levels, which favoured muscle development. According to Cartaxo et al. [36], variations in the energy and protein levels of diets affect the cold carcass weight, and, consequently, increase the CCI in similar animals.

The effect of water supply on spleen weight might be a function of the increased activity of this organ as a result of water deficiency, leading to haemoconcentration due to the increase in haemoglobin levels and blood concentration in response to water loss [21, 37]. Considering that the functions of the spleen are linked to the blood, it is possible that the 48-hour water restriction lead to an increased spleen activity and, ultimately, increased spleen size.

Higher levels of forage cactus silage increased the CP content (14.43%), reduced the aNDFom concentration (39.92%), and increased the NFC participation, contributing to the higher nutritional value of the diet and stimulating liver development, as observed in this research. The liver is important for various metabolic processes and is involved in the energetic and protein metabolism of animals [12], in the uptake of ammonia, and in urea conversion, as well as in amino acid synthesis and degradation. The diets with higher forage cactus levels resulted in increased perirenal fat deposition, 0.32 kg with 42% forage cactus silage compared to 0.19 kg in diets with lower forage cactus levels. Since forage cactus has a higher NFC content than Tifton hay, the higher the addition of cactus silage, the fatter deposition. Consequently, there is a higher availability of precursors for lipogenesis, resulting in fatter deposition. However, perirenal fat represents an energy reserve and has no value for producers. Sheep and goats tend to deposit the perirenal and visceral fat first, then the subcutaneous depositions, and finally the intramuscular depositions [16, 38]

The lack of an effect on live weight at slaughter indicates that the water restriction up to 48 h was not sufficient to reduce feed intake to directly affect body weight. Additionally, according to Qinisa et al. [39], moderate water restriction mainly affects body water loss rather than tissue mass, without deleterious effects on production or growth.

The dietary NFC content was higher for animals fed with cactus silage; this diet, therefore, provided higher energy and nitrogen levels, strongly influencing ruminal digestibility and hence the nutrient fluxes of both volatile fatty acids and proteins [40], resulting in increased nutrient flow to the tissues and favouring muscle anabolism. As a consequence, such animals were heavy, with higher carcass yields. The highest hot carcass yield can be attributed to the higher CCI of the animals fed with 42% cactus silage; those who had larger morphometric measurements and also presented more muscle filling became larger and more compact, and these characteristics are evident in the proportion of muscles and in the larger rib eye area. The lower carcass yield observed for lambs fed without cactus silage could possibly be attributed to a lower carcass weight and higher GIT content. Animals subjected to a diet without cactus silage only received Tifton hay as roughage, which has a higher aNDFom content. As a result, feed passage through the intestinal tract was slower, leading to lower HCY and CCY levels.

The higher energy availability derived from diets containing cactus silage, with a higher availability of nutrients for the development of muscle and adipose tissue, resulted in a higher weight of the half-carcass as well as of all the commercial cuts. Due to the higher values of the body condition scoring in the animals that received cactus silage, we infer that this diet promotes a higher deposition of subcutaneous fat in the carcasses, resulting in lower losses by cooling. According to Sañudo et al. [38], the subcutaneous fat layer forms a blockade that prevents water loss from the carcass.

The larger rib eye area observed in lambs fed with diets containing 42% cactus silage can be explained by the slaughter weight differences associated with the absence of a difference in the croup width, therefore, the animals fed with 42% silage had a higher gain of weight and, consequently, a greater accumulation of muscle in the body that resulted in a greater CCI, which was evidenced in the larger perimeters of the leg and rib eye area [38].

According to Cezar and Sousa [16], the cuts with the highest commercial value are the leg and the loin, denominated prime cuts or first category, since they show greater muscle yield and tenderness. In the present study, higher weights were found for the leg and loin of animals fed with cactus silage, the inclusion of cactus silage promoted an increase in dry matter intake, consequently increased body weight gain and consequently the animals had greater development of these cuts that have a large proportion of muscle.

The rib eye area values were not affected by intermittent water supply of 48 h, however the inclusion of a diet containing 42% cactus silage promoted an increase in rib eye area, it can be inferred that only the diets promoted differences in carcass compactness. The animals being of similar age, size and phenotypic characteristics had larger carcasses and half weights and yields when ingested cactus silage because they had a higher performance.

The animals that received diets without cactus silage and were submitted to intermittent water supply to 48 h presented lower Rib yield, it can be supposed that the water restriction and the higher values of aNDFom and Acid detergent fiber, reduced the passage rate and consequently, affected dry matter intake and thus caused lower performance.

The intermittent water supply had no effect on the leg muscle index and tissue composition, most likely because an intermittent water supply of up to 48 h did not affect the productive performance of lambs, resulting in similar LWS values among the intermittent water supply treatments. Consequently, there were no differences in carcass weight when the animals were submitted intermittent water supply.

The animals fed with cactus silage presented higher muscle and bone weights as a consequence of the higher leg weight. The higher leg weight is most likely a result of the higher carcass weight of animals fed with cactus silage. The higher energy value of diets containing 42% forage cactus resulted in more leg fat, the higher percentage of fat in the leg can be attributed to the higher energy intake, caused by the higher dry matter intake observed also in the higher slaughter weight provided by cactus silage. According to Kioumarsi et al. [41], the simultaneous increase in dietary energy and protein levels in ruminants is related to an increase in amino acids used as pre-glycolytic components in the production of acetate, which can be used to produce acetyl-CoA and, in turn, for the biosynthesis of fatty acids.

This absence of variation in pH 24 h among treatments can be explained by the fact that diet and intermittent water supply did not affect glycogen reserves in the muscle. Thus, its conversion to lactate and H^+^ was similar among the treatments. This content, in turn, also depends on the pre-slaughtering state of animals. Stressed animals partially or completely use their muscle glycogen reserves [42]. In the present study, the animals were not subjected to any pre-slaughter stress, resulting in average pH values after 24 h of 5.68 for diets with cactus silage and average pH values after 24 h of 5.69 for animals subjected to intermittent water supply, which are within the limits (5.4–5.7) reported by Sebsibe [43] for good-quality meat.

Higher levels of cactus silage culminated in heavier animals, i.e., fat deposition was more evident, and hence decreased the water content of the muscle, resulting in a lower luminous intensity [44].

The addition of cactus silage significantly reduced the intensity of the myoglobin and haemoglobin pigments, which was reflected in the intensity of the red (a *), and resulted in a reduction in Chroma, which is directly related to the concentration of myoglobin present in the meat, for which the lower the value the greater the matte colour intensity [45]. The intensity of the yellow (b *) had the same behaviour (a * and Chroma) but is more representative of fat colour, that is, it is related to the accumulation of carotenoids in the fat, such as lutein, which is found in a higher proportion in grasses than in the cactus pear and is stored in the adipose tissue of sheep [46].

The shear force of the meat did not present a significant difference between the treatments, probably due to this parameter being unrelated to the diet and more directly related to the age, breed and sex of the animal, as the animals were of the same sex, of similar race and age, no differences were observed. However, the meats were considered soft, since according to Cezar and Sousa [16], fillets that did not resist a cutting pressure of 2.27 kgf/cm^2^ are considered soft.

Palmquist et al. [47] found that the diet ingested by animals is likely to be the main factor affecting the biohydrogenation process, since dietary changes induced by feed can alter the path of biohydrogenation, resulting in significant changes in intermediate fatty acids. In view of the above, the lack of any effects of intermittent water supply in the present study can be attributed to the similar flow of nutrients from similar diets. The intermittent water supply was not reduced the DM intake, nor was the ruminal environment changed, which not changing the biohydrogenation process. However, diet affected microbial activity as observed in C15:0iso and C15:0anteiso.

Among the SFA, C14:0 (1.85%), C16:0 (24.95%), and C18:0 (16.63%) were the most abundant ones in the lipid profiles in relation to the total saturated fatty acids. Similar profiles have been reported previously [48, 49]. The inclusion of a source of higher carbohydrate concentration (pectin) such as cactus silage promotes greater energy intake and the animal stores this excess energy as fatty acids up to 16 carbons, as described in the literature, fatty acids in meat above 18 carbons are usually derived from the diet [48, 49].

The reduction in C18:0 and C21:0 in the treatment with 42% cactus silage in the diet is desired, since SFA is associated with coronary heart diseases [50]. However, not all saturated fatty acids represent a health risk, and according to French et al. [51], even high levels of stearic acid have no impact on cholesterol levels, since it is poorly digested and easily desaturated at C18:1.

Vlaeminck et al. [52] found that the dietary starch content was negatively correlated with isomyristic acid, iso-pentadecanoic acid, anteiso-pentadecanoic-acid, and isopalmitic acid, whereas there were positive correlations between the aNDFom in the diet and isomyristic acid, iso-pentadecanoic acid, anteiso-pentadecanoic acid, and isopalmitic acid. In view of the above, the higher the ratio of Tifton hay in the diet, the higher the aNDFom levels, resulting in an increased population of cellulolytic bacteria in the rumen. However, the forage cactus presents higher levels of NFC and lower levels of aNDFom, increasing the concentration of propionate in the rumen [53]. The fibrous carbohydrates of Tifton hay produce a higher proportion of acetate when fermented than forage cactus silage, which might explain the higher levels of branched chain fatty acids deposited in the meat fat of lambs fed with diets without forage cactus silage.

The total saturated fatty acids (SFA) were not altered by the intermittent water supply, probably since few SFA were influenced by the substitution, which was not reflected in the total, averaging 45.96%. Due to biohydrogenation, the fatty acids derived from the diet are hydrolysed, and polyunsaturated fatty acids are rapidly hydrogenated by the rumen microorganisms, resulting in saturated fatty acids. This is one of the main reasons for the highly saturated nature of lipids in ruminants [54].

The MUFAs, as well as the SFA, are mainly affected by ruminal biohydrogenation, which in turn can be affected by several factors. The pH decrease alters the ruminal microbiota, influencing the fermentation pattern of the final product [55]. In sheep from the Sudanese desert, Ahmed and Abdelatif [56] observed that animals subjected to water restriction showed reduced ruminal pH levels. However, the similarity between the MUFAs observed with an intermittent water supply of up to 48 h in the present study can be explained by the absence of effects on the ruminal environment, and hence on biohydrogenation.

The C18:1 c9 (oleic acid) was the MUFA with the highest percentage and the greatest contribution to the lipid profile of lamb meat (38.80%), as observed by Sañudo et al. [38], who mention that this acid varies between 30 and 43% in the lipid profile of the meat. It is a hypocholesterolemic fatty acid, which, besides not decreasing the high-density lipoprotein (HDL) levels, prevents cardiovascular diseases by reducing the low-density lipoprotein LDL levels [57].

Higher levels of forage cactus silage in the diet might lead to changes in the ruminal environment, thus modifying biohydrogenation. Consequently, some unsaturated fatty acids from the diet might have been absorbed in the small intestine and incorporated into the meat, as observed for myristoleic acid and gadoleic acid.

The reduced linolenic acid content of the lambs’ diets containing forage cactus silage might be a reflection of the lower content of ether extract in these diets. Thus, the lowest concentration of ether extract in the silage was probably sufficient to reduce the incorporation of α-linolenic acid in the intramuscular fat of the group of animals fed cactus silage in the diet.

There were no effects of the different diets on PUFAs, C18:2 c9t11, and C18:2 t10c12, which are intermediates of biohydrogenation and present the positions and geometry of isomers of linoleic acid with a conjugated double, known as conjugated linoleic acid (CLA) [54]. The CLA results from incomplete biohydrogenation in the rumen, and the absence of differences in its composition might indicate that the diet had no impact on ruminal biohydrogenation. It is noteworthy that ruminant meat is among the natural sources rich in CLA isomers, particularly 18:2c9t11, which is mainly produced in tissues by the action of the enzyme Δ9-desaturase on vaccenic acid (C18:1 Trans-11) and by the ruminal biohydrogenation of unsaturated fatty acids in the diet [58].

The treatment with 0% forage cactus silage presented a higher concentration of n3, which can be explained by the fact that grass-based voluminous, such as Tifton hay, are sources of n3 fatty acids, mainly linolenic acid (C18: 3 n-3) [59], whereas *Opuntia* generally shows a higher concentration of C18: 2 n-6 linoleic acid [60].

Omega 3 is an essential fatty acid, i.e. it is not synthesised by the animal’s body, and therefore, must be provided by the diet to improve the lipid profile of the meat and supply the consumer with a better quality product, since omega 3 fatty acids have several effects on the immune and inflammatory responses. For example, n-3 has suppressive effects, such as the inhibition of lymphocyte proliferation, cytokine production, and antibodies; the expression of adhesion molecules; and the activation of natural killer (NK) cells, which are important for human health [61].

In relation to the nutraceutical parameters that are strongly correlated with the risk of cardiovascular diseases and obesity in humans [62], no significant difference was observed with different levels of palm silage or water availability, however, it was observed that AI and TI presented averages of 0.35 and 0.91, respectively, which are considered ideal because they are below the average recommended by Ulbricht and Southgate [29] for lamb meat that is from 1.00 for AI and from 1.33 to 1.58 for TI. The lower these values in the lipid fraction of the product, the higher the prevention of the early stages of cardiovascular diseases [63].

Arruda et al. [63] studied the relationships or ratios among fatty acids, with the objective to verify and identify the risk factors of food in relation to the increase in blood cholesterol levels in humans. Since the intermittent water supply of up to 48 h did not alter any fatty acids, their ratios and indices were also not influenced, indicating that no parameters of the lipid profile of the lamb meat were changed.

Based on the observed results, crossbreed lambs show efficient adaptation mechanisms to an intermittent water supply, especially when receiving cactus silage; these energy-rich diets resulted in lower feed conversion, increased weight gains, and consequently, positive effects on feed conversion, average daily gain, carcass yield, and the meat quality of sheep fed diets with higher levels of cactus silage. These results are relevant since the use of a high ratio of cactus silage in sheep diets, besides increasing carcass yield and improving meat quality, can reduce the water demand of the animals, which is an important factor in semiarid regions.

## Conclusion

The substitution of Tifton hay by 42% forage cactus silage is recommended, since it provides higher carcass yields, non-carcass constituents, and commercial cuts, besides increasing the meat quality of lambs.

An intermittent water supply of up to 48 h does not influence carcass characteristics and meat quality. Therefore, the use of cactus silage can be recommended in situations of water scarcity without harming the production and meat quality of crossbreed lambs.
